# Water, sanitation, and hygiene (WASH) facilities and infection control/prevention practices in traditional birth homes in Southwest Nigeria

**DOI:** 10.1186/s12913-021-06911-5

**Published:** 2021-09-03

**Authors:** Adediwura Oladunni Arowosegbe, David Ajiboye Ojo, Olufunke Bolatito Shittu, Opeoluwa Iwaloye, Uwem Friday Ekpo

**Affiliations:** 1 Department of Microbiology, College of Biosciences, Federal University of Agriculture, Abeokuta, Nigeria; 2 Department of Zoology, College of Biosciences, Federal University of Agriculture, Abeokuta, Nigeria

## Abstract

**Background:**

Poor environmental conditions and hygiene practices at the time of childbirth is linked to life-threatening infections and death in mothers and babies. Improvements in water, sanitation, and hygiene (WASH) have been identified as a means through which the lives of mothers and babies could be saved. This study was carried out to explore WASH conditions and infection prevention and control (IPC) practices in traditional birth homes/centers in Abeokuta, Southwest Nigeria. A total of 50 traditional birth centers and attendants (TBAs) were enrolled in the study. Sociodemographic characteristics of the TBAs and features of the birth centers were obtained using a semi-structured questionnaire. Assessment of WASH conditions and IPC practices was based on established protocols.

**Results:**

Findings revealed that majority of the centers operated under poor WASH conditions and IPC practices; none met with the WHO minimum standards for environmental health.

**Conclusions:**

Adequate WASH facilities and IPC practices remain a critical component of maternal and child health even in non-facility birth. As the transition to facility births continues in many countries, the large number of non-facility births demands their inclusion in WASH-related strategies, if global goals of reducing deaths of newborns and women deaths will be achieved.

## Background

Although clean water has received the most attention, the greatest health benefits and increase in standards of living are achieved when improvements in sanitation and hygiene are made alongside access to clean water [1, 2]. Until recently, water, sanitation and hygiene (WASH) interventions have largely focused on households and communities [3, 4], however, WASH and infection prevention and control (IPC) practices in healthcare facilities (HCF) is gaining more attention [5, 6]. Adequate WASH services and maternal-neonatal health, two related, crucial subjects, are now targets of the Sustainable Development Goals [7].

The links between hygiene and healthcare-associated infections in mothers and their babies have been established [8–10]. Clean delivery practices are associated with decreased sepsis and tetanus neonatal mortality [11]. However, in many parts of the world, and especially in Africa, substantial proportion of women deliver at home and in health facilities deliver without access to essential WASH services that ensure clean practices [8, 12, 13]. These gaps are more pronounced in rural areas with smaller facilities than in larger facilities in urban areas [8, 12]. While working towards increased uptake of skilled attendance, many sub-Saharan Africa countries have continued to work with traditional birth attendants (TBAs) as “interim” partners in maternal and infant health care. A large percentage of births in LMICs are attended to outside health facilities by TBAs [14–16] without requisite clean delivery tools leading to even greater chances of infection [17].

Compared to efforts on health system strengthening, and increased uptake of facility births [18, 19], less focus has been paid on environmental conditions during delivery. Poor WASH has major health and survival implications for mothers and their babies. The risk of infection from poor hygiene contributes to life-threatening infections in mothers and newborn [20] and remains high not only in healthcare facilities but also in the community [21]. Globally, infection accounts for at least 9 % of maternal deaths [22] and 16 % of neonatal deaths [23]. The highest mortality occurs in low- and middle-income countries (LMICs) [11] and an estimated 700 neonatal deaths [17] and 145 maternal deaths each day [15] in Nigeria.

Despite the burden of maternal and neonatal infections and the links with poor hygiene, data on clean delivery practices around birth or the status of prerequisite facilities necessary for these practices are limited. Beyond health facilities, there is paucity of information on WASH conditions and adherence to clean delivery practices in non-facility births. It is therefore imperative that strategies that are been developed to reduce maternal and neonatal mortality due to preventable causes take into consideration, deliveries that take place in traditional birth centers or homes. The objective of this study was to describe the status of WASH services as well as clean delivery practices in traditional birth (TBHs). The large number of deliveries occurring in these settings necessitates understanding how environmental contexts surrounding these deliveries potentially impact maternal and newborn outcomes and setback global maternal-newborn goals.

## Methods

### Study location

The study was carried out in Abeokuta, the largest city and capital of Ogun State. Its geographical area covers Abeokuta South and parts of Abeokuta North, Obafemi Owode, and Odeda Local Government Area. In Abeokuta, healthcare is provided by few governmental and private hospitals, primary healthcare centers, as well as a myriad of community, faith-based, herbal, and traditional practitioners. Traditional Birth Attendants (TBAs) commonly provide perinatal care in rural and low-income urban areas of the town. TBAs form traditional birth attendant association and are recognized by the government. The activities and practices of the TBAs are regulated by the Primary Health Care Development Board of the State.

### Data/Sample collection

A mixed-methods approach was used to assess WASH services and clean delivery practices in the recruited traditional birth homes (TBHs) (Table 1). The tools used for this assessment were adapted from the WHO/UNICEF Joint Monitoring Program for WASH [24], WHO essential environmental health standards [25], and the WASH & CLEAN toolkit developed by the Soapbox Collaborative to assess IPC on maternity units [26].

**Table 1:** Data collection methods

Data Collected	Approach	Tools	Participant
Status of WASH facilities	Semi -structured Interviews	Questionnaire	TBA
Clean delivery practices	In-depth assessment and walkthrough technique	Walkthrough checklist, Microbiological analysis	TBA

First, in-depth interviews were conducted with TBAs in their birth homes using a semi-structured questionnaire as a guide. The questionnaire was used to assess the status of WASH facilities and clean delivery practices (six cleans) in the birth homes. For assessing the status of WASH services, the following domains were covered: basic water supply, basic sanitation, waste management, and access to basic hygiene in the TBHs (Table 2).

**Table 2:** WASH Domains and Definitions according to WHO and WHO/UNICEF Guidelines

Domain	Features Checked	Recommendation
Water	Water Quality, Water Access	Water from an improved source. Less than 1 *E. coli*/ thermotolerant total coliforms per 100 ml of water.On-site supply
Sanitation	Excreta Disposal, Wastewater disposal, Healthcare waste disposal	Improved sanitation facilities. Provision of adequate, accessible and appropriate toilets for patients, staff, and carer. Wastewater is disposed of rapidly and safely. Health-care waste is segregated, collected, transported, treated, and disposed of safely.
Hygiene	Handwashing, Sterilization	Provision of reliable water-point with soap or alcohol-based hand rubs available in all treatment areas, waiting rooms, and near latrines for patients and staff.Sterilizing equipment or supplies

Assessment of clean delivery practices was based on adherence to WHO’s six cleans including clean hands, clean perineum, clean delivery surface, clean cord cutting, clean cord tying, and clean cord care. Adherence to six cleans was recorded through observation and recording in the walkthrough checklist as well as the use of a questionnaire.

Secondly, an in-depth assessment was combined with the walkthrough methodology, which consisted of recording observations in a checklist and collecting water samples for microbiological analysis. This assessment focused on visual and microbiological cleanliness of birth home environments and tools. Data was collected through observation and recording in the walkthrough checklist. Samples were collected from delivery surfaces and cord-cutting instruments as well as from water used for domestic purposes such as hand washing and bathing in the TBHs

Water quality testing was conducted in all the TBHs. Samples of water available in birth homes were collected directly from sources (on-site) or storage vessels to assess the microbial quality of water used for domestic purposes and during delivery.

### Data analysis

#### Interviews

Analyses of the TBHs interviews focused on the description of WASH facilities and clean delivery practices. Data analysis was carried out using the Statistical Package for Social Sciences (SPSS) software (version 17.0). Descriptive statistics (including frequencies, percentages, charts, and tables) was used to describe WASH conditions and clean practices

#### Walkthrough checklist

Visual Inspection and Microbiological Analysis:

The analysis of both the checklist and microbiological data focused on describing the cleanliness at different sites of the birth home as well as cleanliness of birth tools/equipment and the cleanliness at the different sites of the birth homes. Isolation of microorganisms from water samples was carried out on general-purpose and selective media using the membrane filtration methods. Microbiological analysis of water samples focused on the presence of Enterococcus and fecal coliforms; standard indicators for assessing water quality. Total bacterial count in the water samples was also determined.

## Results

### Characteristics of traditional birth homes and birth attendants

A total of 50 traditional birth/herbal homes (TBHs) were recruited through the State Birth Attendant Association into the study. Only one-fifth of the traditional birth homes were solely for maternal and newborn care, others were into other forms of herbal or general healthcare. An average of one - five deliveries in a month was reported from the homes. Many (58 %) of the homes were operated by the birth attendants with the support of family members (Table 3). TBAs were mostly older females having more than 10 years of work experience in most cases (Table 4).

**Table 3:** Features of Traditional Birth Centres included in the Study

**Features**	**Variables**	**Frequency (N)**	**Percentages (%)**
**Local Government Area**	Abeokuta North	29	43.3
	Abeokuta South	34	50.7
	Odeda	03	4.5
	Obafemi Owode	01	1.5
**Type of Health Service**	Perinatal	16	23.9
	General	51	76.1
**Type of TBC**	Community-based	39	58.2
	Faith-based	13	19.4
	Herbal Practitioners	15	22.4
**Average number of Delivery**	1–5	50	73.1
	5–10	09	13.4
	11–15	07	10.4
	16–20	01	1.5
	20	03	4.5
**Number of Staff**	1–5	53	79.1
	5–10	11	16.4
	11–15	01	1.5
	16–20	01	1.5
	20	04	5.9
**Type of Staff**	Family	39	58.2
	Trainee/Apprenticeship	28	41.8

**Table 4:** Sociodemographic Characteristics of the Traditional Birth Attendants in the Study

**Features**	**Variables**	**Frequency (N)**	**Percentages (%)**
**Sex**	Male	12	17.9
	Female	45	82.1
**Age**	20 – 30	05	07.5
	30 – 40	15	22.4
	40 – 50	26	38.8
	>50	21	31.3
**Educational Level**	None	20	29.9
	Primary	19	28.4
	Secondary	28	41.8
**Source of Skill Acquisition**	Apprenticeship	37	55.2
	Family	21	31.4
	Semi-Formal Training	09	13.4
**Subsequent Training**	None	36	53.7
	1–5	11	16.4
	>5	20	29.9
**Years of Practice**	1–5	05	07.5
	5–10	13	13.4
	11–15	21	31.3
	16–20	08	11.9
	20	20	29.9
**Other Profession**	Yes	39	58.2
	No	28	41.8

### Status of WASH in TBHs

#### Water quality, quantity, and access

**Fig. 1:**
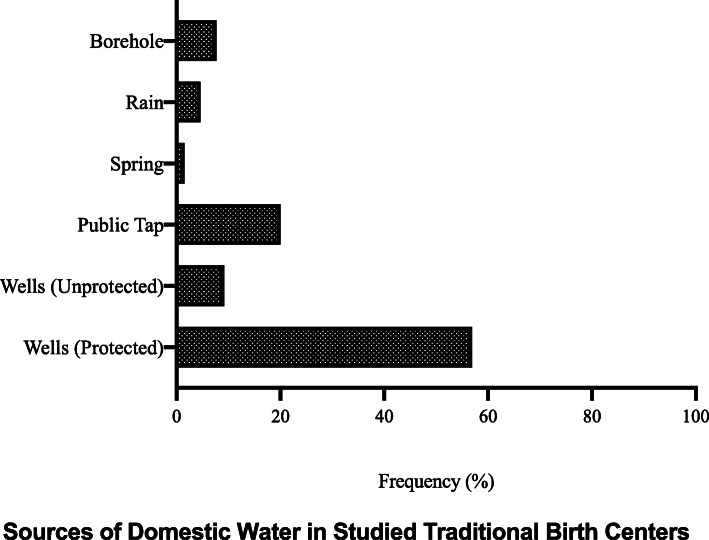
Sources of domestic water available at Traditional Birth Centres

Uncovered wells were the most common source of water in the TBHs. Other sources included springs, boreholes, rain, covered well, and public tap water. Some birth homes (36 %) relied on two or more sources. Unimproved water sources were the most common (66 %), followed by improved sources (22 %) and a combination of both sources (22 %) (Fig. 1). 64 % of the birth homes did not have their main water source located on the premises and depended on water sources in nearby homes or on water supply from community taps. Furthermore, access to consistent, improved water supply was found in only 12 (17.9 %) of the birth homes. Of the 12 birth homes with access to improved water supply, only 6 % had consistent running water in the delivery room. Lack of reliable supply of water was supplemented for by storage of water in all the birth homes (Table 5).

**Table 5:** Access to Water Supply in Studied Traditional Birth Centres

Water Access	Frequency (%)	Category	Frequency (%)
On-site	24 (35.8 %)	Improved Unimproved Both	12 (17.9 %) 05 (7.4 %) 07 (10.4 %)
Not on-site	43 (64.2 %)	Improved Unimproved Both	02 (3.0 %) 36 (53.7 %) 05 (7.5 %)

**Fig. 2:**
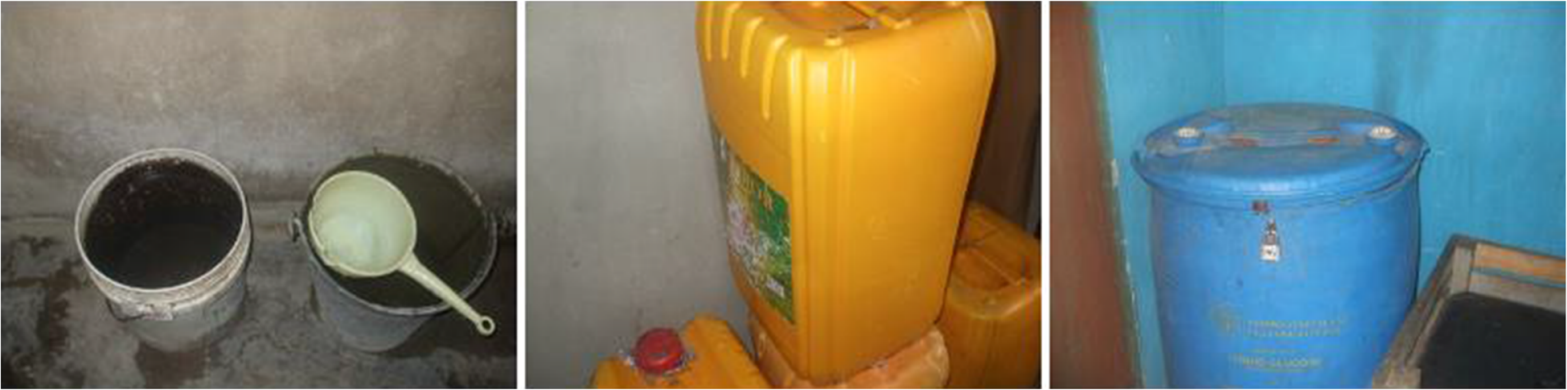
Water Storage in some of the Traditional Birth Centers

**Fig. 3:**
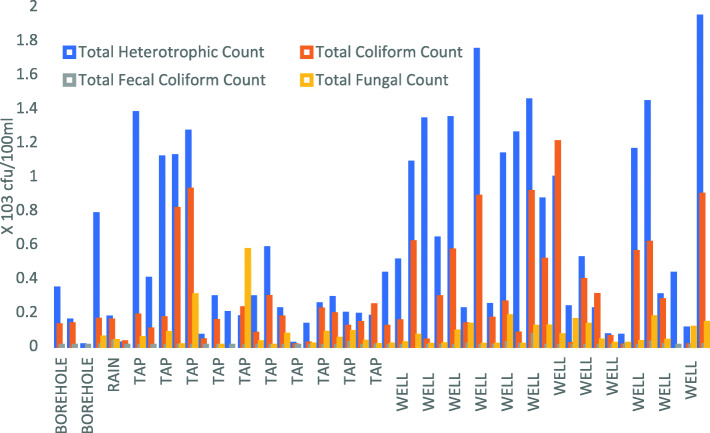
Bacterial Counts in Water Samples from Studied Traditional Birth Centres

Walkthrough assessment collaborated data from the interview and revealed inadequacies in water storage as storage containers in many of the TBHs were visibly soiled. Stored water was visibly contaminated with debris or a film on the surface (Fig. 2). In addition to the supply of water, the quality of the water was also found to be a major issue. The results show that of the 50 water samples collected from the birth centers, the presence of fecal coliform was detected in 22 % of the birth centers. Most (88 %) of the water samples from the birth centers had very high bacterial counts of over 100 colony forming units (CFU)/100ml indicating heavy contamination of the water samples (Fig. 3; Table 6). Well water had the most bacterial counts. All stored water had very high bacterial counts over 100 colony-forming units indicating poor storage conditions.

**Table. 6:** Bacteriological and Fungal quality of Water Samples

	Range (CFU/ 100mL)	Mean ± SE (CFU/ 100mL)	Median (CFU/ 100mL)	WHO Standard
Total Bacterial Count	0.035– 19.4 × 10^4^	5.99 ± 0.76 × 10^4^	3.19 × 10^4^	100 cfu/ml
Total Coliform Count	0.00–12.0 × 10^4^	2.72 ± 0.41 × 10^4^	1.62 × 10^4^	0
Total Fecal Coliform Count	0.00–6.5 × 10^2^	3.82 ± 1.51 × 10^2^	0	0
Total Fungal Count	0.00–5.3 × 10^3^	5.98 ± 1.35 × 10^3^	2.35 × 10^3^	NA

### Sanitation

#### Excreta disposal

**Fig. 4:**
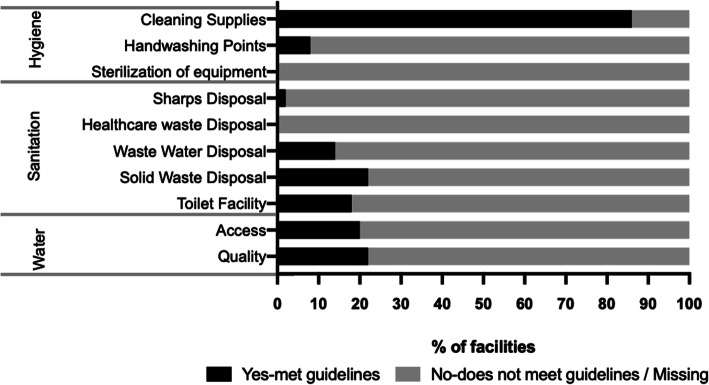
WASH Facilities in enrolled Traditional Birth Centers

**Fig. 5:**
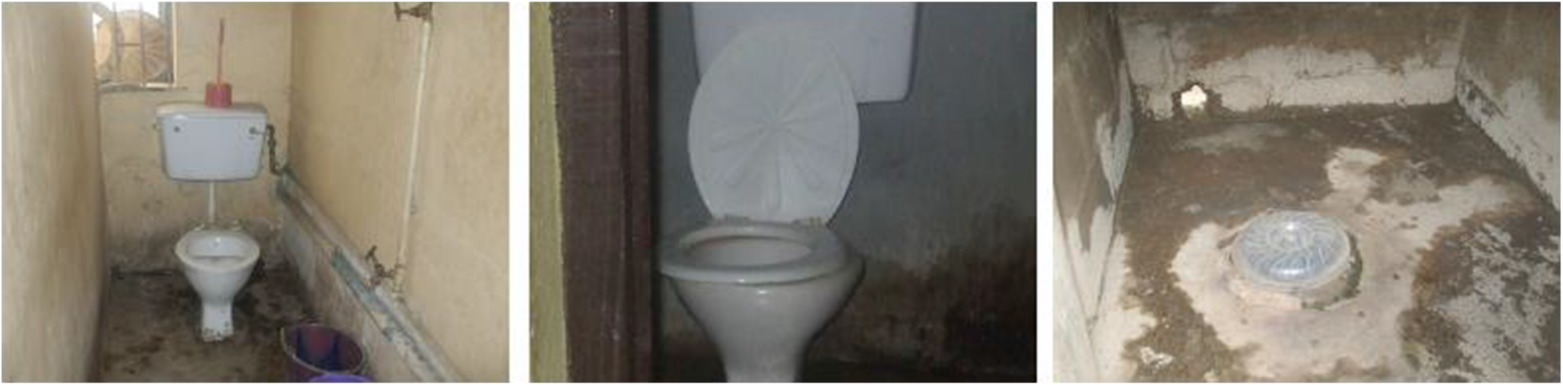
Toilet Facility in some of the Traditional Birth Centers

About two-thirds of the birth homes (68 %) had no toilet facility reserved for use by TBAs and women in delivery. Access to improved toilet facilities was found in 34 % of the centers (Fig. 4). Where present, walkthrough assessment revealed poor sanitary conditions of toilets and the absence of cleaning materials (Fig. 5). Standard protocols for solid waste disposal and wastewater disposal were missing in 78 and 84 % of the birth centers respectively (Fig. 4).

There were no provisions for sharps disposal in any of the birth homes. None of the centers had a standard protocol for healthcare waste disposal (Fig. 4).

### Hygiene

**Fig. 6:**
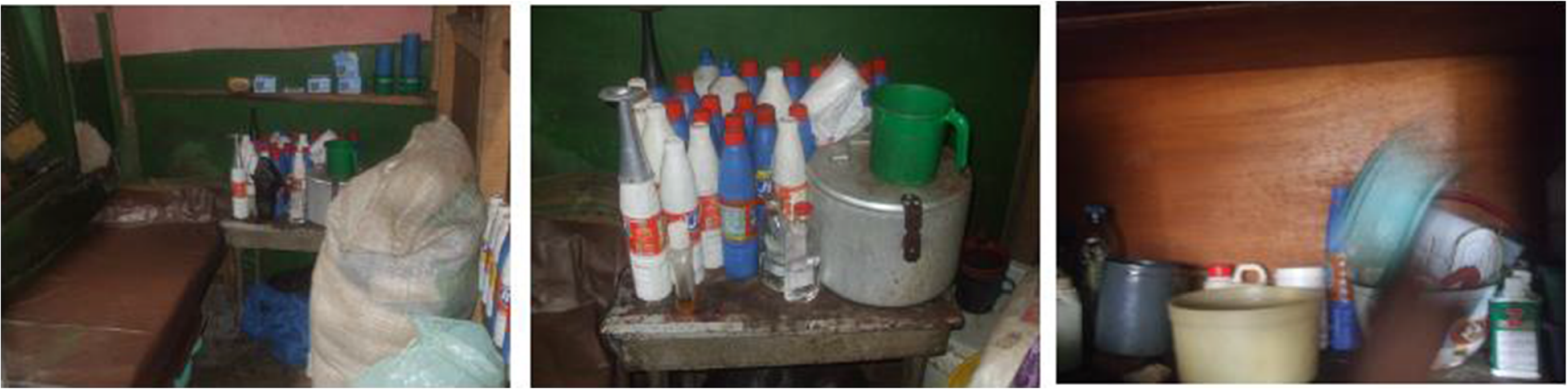
Cleaning agents and Disinfectants in some of the Traditional Birth Centers

Reliable handwashing points with reliable water and soap was found in only 8 % of the delivery rooms (Fig. 4). All birth homes reported the use of cleaning supplies, walkthrough assessment of birth homes showed that basic cleaning supplies including detergents and or bleach were found in 86 % of the delivery rooms (Figs. 4 and 6).

### Clean delivery practices

**Fig. 7:**
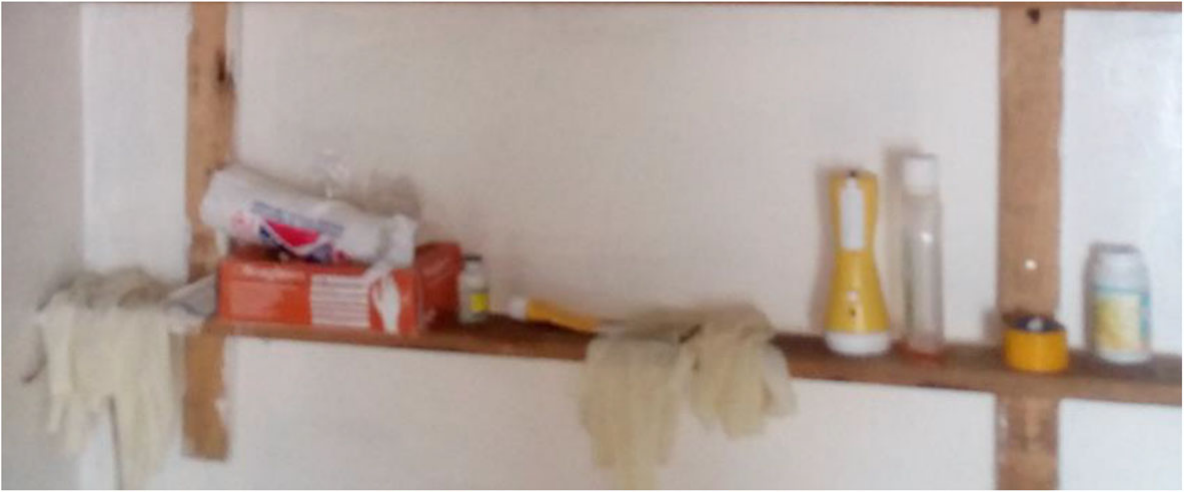
Storage of single-use Gloves in one of the Traditional Birth Centers

Clean Hands: All TBAs reported the use of gloves for vaginal exams or when handling the baby. However, correctness of glove use was not established among any of the TBAs. There was also a lot of negligence on the correct use of gloves including re-use of gloves and improper storage of gloves (Fig. 7).

**Fig. 8:**
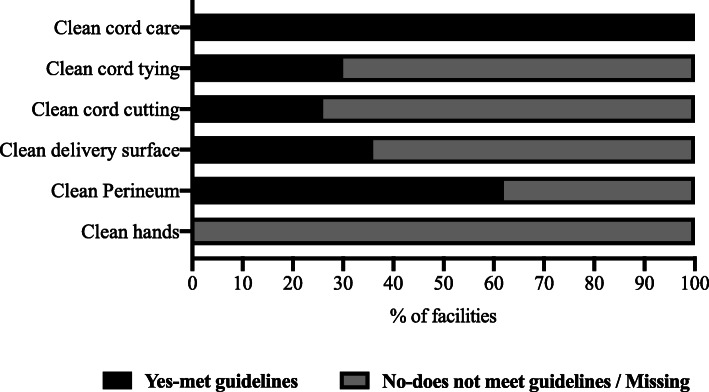
Clean Delivery Practices in Traditional Birth Centers

Clean Hands: Although handwashing was reported by 76 % of the TBAs, adherence to WHO recommendations on the five moments of hand hygiene was not reported or described by any of the TBAs. Other TBAs regarded glove use as synonymous to handwashing. None of the TBAs met established guidelines for clean hands (Fig. 8).

Clean perineum: Assessing of clean perineum was limited to insertion of herbs because TBA inconsistencies with perineal cleaning and cofounding by patient perineal cleaning. Insertion of herbs during delivery was reported by 38 % of the TBAs (Fig. 8).

Clean delivery surface: All TBAs reported cleaning of delivery surfaces after each delivery but not routine cleaning. 36 % of the TBAs reported the use of disposable bed overlay during each delivery. Walkthrough assessment revealed dusty, poorly cleaned, and worn-out delivery surfaces.

Clean cord-cutting: Single-use razor blade was reported by 26 %, 46 % use scissors while 28 % use both. Sterility of scissors used in cord severance was debatable because there were no standard protocols for sterilization of delivery instruments (Fig. 8).

Clean cord tying: Use of sterile cord clamps was reported by 14 % of the TBAs. Other TBAs (86 %) reported the use of ties made from threads and clothes; 16 % of these TBAs soaked cord ties in methylated spirit (Fig. 8).

Clean cord care: Following cord cutting, TBAs reported the use of either methylated spirit (72 %), chlorhexidine gel (12 %), or both (16 %) in cord care (Fig. 8).

## Discussion

Availability of adequate WASH services and hygiene practices during births are acknowledged as key to ensuring better outcomes during childbirth [27–29]. The calls for WASH strategies to be included in global and national strategies [30] require evaluation of WASH status across facilities to drive policies and strategies [30]. This study provides an illustrative analysis of WASH services and clean delivery practices in non-facility based maternity centers run by TBAs in a poor-resource setting. Even amid transition to skilled birth attendance at births, TBAs remain key partners in maternal care and the large number of deliveries occurring outside healthcare facilitates necessitates inclusion of these births in strategies that seek to reduce mortality.

Data from our study across the 50 traditional birth homes highlights gaps in WASH services and clean delivery practices. Findings revealed majority of the birth homes operated under poor WASH conditions. None of the birth centers matched up with the WHO minimum standards for environmental health conditions with regards access to safe and sufficient WASH facilities [25].

First, there was alarming lack of reliable and continual water supply, with majority of the centers lacking access to improved water sources. More than half of the centers had no sanitation facilities for TBAs and mothers’ use. None of the centers adhered to standard protocols on healthcare waste disposal and sterilization. Functional handwashing points were absent in majority of the birth homes. Interviews with TBAs and walkthrough observations also revealed major deficits in TBA knowledge and practice of clean delivery practice for the various components of the six cleans, especially for clean hands, clean perineum, and clean delivery surfaces.

Results from this study are not unexpected and corroborate previous reports on the alarming lack of basic WASH services in healthcare facilities in different communities across Nigeria [31]. In a study carried out to assess 242 healthcare facilities, 7.85 % have no form of water source and 22 % had no toilet facility. Handwashing facilities were observed in the delivery rooms of only 54.9 % of PHCs assessed. Another study identified poor hand hygiene as a barrier to delivering safe care in health facilities [32]. These observations are also supported by studies from other LMICs [13, 33, 34]. In one of these studies carried out in Zanzibar, for every of the ‘cleans’ examined, overall performance across all enabling factors was poor [34]. Sadly, little progress is made in improving these conditions in Nigeria. A recent study reveals that the readiness to provide routine maternal and newborn care in sampled health facilities in Nigeria fell from 25–0 % while that of two other LMIC, Ethiopia and Uttar Pradesh increased [35].

The gaps in WASH and clean delivery practices in these traditional birth homes preclude optimal hygiene during delivery. Suboptimal WASH conditions and ICPs predispose and increase the vulnerability of women and their newborns to the risk of life-threatening infections. Although findings from the study are unsurprising due to limited resource availability and accessibility in low-income settings, they should top consideration in strategies that seek to improve newborn and maternal health at the community level.

The Nigeria government has several WASH development strategies to ensure provision of safe and accessible WASH services for all of its citizens, however, many of these strategies have failed to translate into actionable plans [36]. Also, they are largely focused on water, leaving behind other components of WASH. There is also the need to close the gaps between WASH strategies and healthcare organizations. Leading agencies responsible for the WASH sector at national and state levels such as the Federal Ministry of Water Resources and State Water Boards could play active roles in ensuring that beyond catering to communities, WASH strategies directly impact healthcare settings. An agreed definition of WATSAN in maternities would also enhance standardized monitoring and this has been exemplified in JMP’s definition for home WATSAN [8].

Improved household WASH condition is definitely a plus. Since many of these births with TBAs take place in home environments, improving the quality of WASH facilities in communities will directly impact these births. Clean births can only achievable in birth environments where the most basic level of improved water and sanitation access are present [13]. Efforts on WASH that focus solely on health facilities will be rendered ineffective by gaps present in the community.

The state of WASH in all healthcare settings need to be continuously monitored to track decline or improvement. It is known that improvements in WASH facilities are not all that is needed [32], therefore, in addition to exploration of WASH status, other efforts such as training and compliance assessment are needed. Research studies that would help suggest associations between lack of, or inadequate, WASH facilities, and particular outcomes, such as maternal and newborn mortality, birth outcomes, and neonatal morbidity will be of great value in the field.

## Conclusions

Increased availability of skilled birth attendants and a reduction in the demand for TBA services remain key strategies for improving newborn and maternal health across the globe. However, findings from this study draw attention to the need to continuously engage TBAs as important interim partners in maternal health care as the transition to skilled attendance continues. Addressing unsanitary conditions during delivery in TBHs is important in avoiding infection-related deaths and achieving global reduction in maternal and neonatal mortality rates. Appropriate policies and protocols should be instituted to ensure infection prevention and control in all maternal health care settings including non-facility settings. Enlightenment and education on clean delivery practices within the communities would also be a valuable tool in reducing maternal morbidity and mortality in these settings.

## Limitations

This study was limited in a number of ways. First, the study included only TBAs registered with the TBA association in the capital city of the State. These TBAs do not completely represent untrained birth workers in the state. They are recognized by the state government and may benefit from training programs organized by the government or other NGOs. It is also likely that there is more awareness on general health and wellness unlike other parts of the state. Therefore, the results identified from the study may not entirely reflect what obtains in other geographic regions beyond the area studied, especially in the rural areas where TBA care for most of the pregnancies and delivery.

The study does not cover practices during the postnatal period in the birth homes which is also a critical time in maternal and neonatal mortality. Although interviews were supplemented with walkthrough observations, some of the assessments may be subject to recall bias as they depended on the ability of TBAs to recall and report on WASH facilities as well as clean delivery practices.

## Data Availability

All data generated or analysed during this study are included in this published article.
